# Nutrient Limitation of Native and Invasive N_2_-Fixing Plants in Northwest Prairies

**DOI:** 10.1371/journal.pone.0084593

**Published:** 2013-12-27

**Authors:** Andrea S. Thorpe, Steven Perakis, Christina Catricala, Thomas N. Kaye

**Affiliations:** 1 Institute for Applied Ecology, Corvallis, Oregon, United States of America; 2 United States Geological Survey, Forest and Rangeland Ecosystem Science Center, Corvallis, Oregon, United States of America; Portland State University, United States of America

## Abstract

Nutrient rich conditions often promote plant invasions, yet additions of non-nitrogen (N) nutrients may provide a novel approach for conserving native symbiotic N-fixing plants in otherwise N-limited ecosystems. *Lupinus oreganus* is a threatened N-fixing plant endemic to prairies in western Oregon and southwest Washington (USA). We tested the effect of non-N fertilizers on the growth, reproduction, tissue N content, and stable isotope δ^15^N composition of *Lupinus* at three sites that differed in soil phosphorus (P) and N availability. We also examined changes in other Fabaceae (primarily *Vicia sativa* and *V. hirsuta*) and cover of all plant species. Variation in background soil P and N availability shaped patterns of nutrient limitation across sites. Where soil P and N were low, P additions increased *Lupinus* tissue N and altered foliar δ^15^N, suggesting P limitation of N fixation. Where soil P was low but N was high, P addition stimulated growth and reproduction in *Lupinus*. At a third site, with higher soil P, only micro- and macronutrient fertilization without N and P increased *Lupinus* growth and tissue N. *Lupinus* foliar δ^15^N averaged −0.010‰ across all treatments and varied little with tissue N, suggesting consistent use of fixed N. In contrast, foliar δ^15^N of *Vicia* spp. shifted towards 0‰ as tissue N increased, suggesting that conditions fostering N fixation may benefit these exotic species. Fertilization increased cover, N fixation, and tissue N of non-target, exotic Fabaceae, but overall plant community structure shifted at only one site, and only after the dominant *Lupinus* was excluded from analyses. Our finding that non-N fertilization increased the performance of *Lupinus* with few community effects suggests a potential strategy to aid populations of threatened legume species. The increase in exotic Fabaceae species that occurred with fertilization further suggests that monitoring and adaptive management should accompany any large scale applications.

## Introduction

Conservation of endangered species often depends on active management of critical habitats and ecosystems [1]. However, management of remnant habitats to support native species is frequently complicated by the overlap in plant traits (for example, phenology, life history, or tolerance to fire or grazing) between native and exotic species [2], [3]. In the Pacific Northwest (USA), one of the primary targets for restoration is *Lupinus oreganus* A. Heller (Kincaid's lupine, Fabaceae; hereafter ‘*Lupinus*’), a federally threatened species. This species is endemic to upland prairies in western Oregon and southwest Washington. In addition to habitat loss, habitat degradation by invasive plants poses one of the greatest threats to this species [4]. Identifying effective management techniques for *Lupinus* is of particular concern as it is the primary larval host plant for the endangered butterfly, *Plebejus icarioides fenderi* Macy (Fender's blue butterfly, Lepidoptera: Lycaenidae).

Plant growth in most terrestrial ecosystems is nitrogen (N) limited [5], [6], but some plants, including those in the Fabaceae family, form symbioses with bacteria that permit fixation of atmospheric N_2_ gas into plant-available forms. Nitrogen fixation, in turn, can be limited by the high phosphorus (P) requirements of symbiotic N-fixing species, or by micronutrients such as molybdenum (Mo) that are essential to the nitrogenase enzyme that fixes atmospheric N_2_ into ammonia [7]–[10]. In agricultural settings, P fertilization has been shown to increase the success of legumes [11]–[13], although there can be great variability in response to fertilization across species [14], [15] and genotypes [16]. While less is known from natural systems, legumes there have also generally been found to have a positive response to P fertilization [17]–[20]. Comparatively fewer studies report evidence of Mo limitation of legumes, though as with P, Mo limitation responses can be species- and site-specific [21]. Molybdenum deficiency can occur in agricultural soils of western Oregon, particularly on acidic hill soils surrounding the floor of the Willamette Valley [22].

Almost 10% of the invasive species in North America are members of the Fabaceae [23]. These species are typically associated with low-nutrient and/or disturbed ecosystems, and their invasion success is linked to their ability to alter nutrient cycling and ecosystem dynamics via N-fixation [20], [24]–[27]. The majority of research on invasive N-fixers has been conducted on woody perennial legumes. Less is known about the effects of herbaceous invasive legumes, though it is likely that their effects differ from those of woody species [28].

The objectives of our study were to determine if P or other non-N nutrients limit the growth of native *Lupinus*, whether these nutrients affect abundance of non-native N-fixers, and whether changes in N fixation can explain these responses. Non-native herbaceous legumes are also common in our study region, but tend to only dominate recently disturbed habitats (e.g. roadsides and recently cleared fields) and are not usually considered significant competitors of native *Lupinus* in remnant and restored prairies. We did not include N fertilization among our experimental treatments out of concern that non-N-fixing species would outcompete these remnant federally threatened populations of *Lupinus*. Previous N fertilization studies have observed losses of diversity and reduction in legume abundance in mixed communities containing symbiotic N fixers [19], [29]. We hypothesized that non-N fertilizers would increase the growth of *Lupinus* and other N-fixing species, but have no effect on non-N-fixing species in these communities. Although sites were initially selected with the intent of being replicates of each other, we hypothesized that there would be differences in soil nutrient status and species responses between the sites due to differences in historic and current management.

## Materials and Methods

### Study sites and treatments

We established three study sites in remnant patches of upland prairie habitat in the Willamette Valley in western Oregon, USA. This region has a seasonal quasi-Mediterranean climate with cool wet winters and warm dry summers. Experimental plots were established in *Lupinus* populations at Baskett Butte (near Dallas; research permission from US Fish and Wildlife Service, Willamette Valley Refuges), Lupine Meadows (near Philomath; research permission from Greenbelt Land Trust), and Wren (research permission from a private landowner) in summer 2006 (See Figure S1 for a map of site locations). Baskett Butte is located on the Baskett Slough National Wildlife Refuge. Although it is likely that this area was at one time grazed by sheep and/or cattle, it has been more than 45 years since this activity occurred. Current management consists of occasional fall mowing. Since 2004, Lupine Meadows (owned and managed by the Greenbelt Land Trust) has been managed primarily for conservation purposes. Prior to entering into a conservation easement, the property was managed as a low-utilization horse pasture and private family recreation area for about 20 years. The Wren population is located on private property and has a history of light grazing and occasional mowing; however, these activities have not occurred for greater than 10 years. All sites are located on Mollisols and differ in surface (0–10 cm) mineral soil carbon (C), nitrogen (N), C:N, and available P (phosphorus) (Table 1).

**Table 1:** Soil characteristics in control plots at the study sites.

Site	Soil Type (USDA)	Soil C (%)	Soil N (%)	Soil C:N	NH_4_^+^ (mg/kg)	NO_3_^−^ (mg/kg)	Net N min (mg/kg−28d)	Bray-P (mg/kg)	pH (H_2_O)
Lupine Meadows	Ultic Argixerolls	3.43^a^ (0.05)	0.23^a^ (0.002)	15.2^a^ (0.78)	1.03 (0.15)	0.00 (0.00)	−1.4^c^ (0.20)	2.86^a^ (0.18)	6.4 (0.1)
Baskett Butte	Ultic Haploxeroll	3.41^a^ (0.18)	0.29^b^ (0.010)	11.6^b^ (0.29)	1.62 (0.62)	0.03 (0.03)	4.3^a^ (2.08)	1.97^b^ (0.14)	6.6 (0.3)
Wren	Ultic Argixerolls	4.23^b^ (0.11)	0.32^b^ (0.007)	13.3^c^ (0.57)	1.73 (0.31)	0.01 (0.01)	−0.1^b^ (0.98)	1.84 ^b^ (0.15)	6.5 (0.1)

Notes: Soil type data from NRCS Soil Surveys for Polk and Benton Counties. Soil chemical variables reflect means of five control plots sampled to 10 cm depth, with standard errors in parentheses. Site differences in chemical variables were analyzed using one-way ANOVA. Values in columns followed by different letters are significantly different (p<0.05) by Tukey post-hoc comparison.

At each site, large areas containing *Lupinus* were mapped onto a grid of 1 m×1 m cells. Cells with either no *Lupinus* or greater than 80% cover of *Lupinus* were rejected as potential treatment plots. From the remaining cells, we selected 20 as treatment plots, with a 1 m buffer surrounding each plot, and staggered the plots to further minimize nutrient movement among them. We used a fully factorial design of two levels each (present/not present) of two fertilizer amendments consisting of phosphorus-only (P) and other non-nitrogen and non-phosphorus macronutrients and micronutrients (M), for a total of four treatments (control, P, M, M+P). Each treatment was replicated five times at each of the three sites. The P treatment was added as super triple phosphate (10 g P m^−2^ yr^−1^). The M treatment was added as potassium chloride (10 g K m^−2^ yr^−1^), calcium chloride (10 g Ca m^−2^ yr^−1^), magnesium sulfate (5 g Mg m^−2^ yr^−1^, 6.75 g S m^−2^ yr^−1^), sodium molybdate (0.02 g Mo m^−2^ yr^−1^), and the commercial nutrient blend Granusol (0.168 g Mn m^−2^ yr^−1^, 0.168 g Zn m^−2^ yr^−1^, 0.168 g Fe m^−2^ yr^−1^, 0.05 g Cu m^−2^ yr^−1^, and 0.017 g B m^−2^ yr^−1^); Granusol was crushed into a powder and all M fertilizers were mixed prior to application Fertilization levels were chosen to largely follow [30]. Each annual fertilization was split evenly into early winter (November/December) and spring (March) applications for 4 years, from November 2006 to March 2009.

### Plant community responses

Measurements of plant cover and composition occurred in May or June of each year, during peak flowering of *Lupinus*, and before the onset of summer drought. In early June 2006, prior to initial fertilization, we measured *Lupinus* cover and counted the number of leaves, and mature and aborted inflorescences. In 2009, we repeated these measurements and also estimated the areal cover of all vascular plant species, as well as mosses/lichens, bare ground, and rock, in all plots. We were not able to sample biomass due to the protected status of *Lupinus* and presence of the endangered Fender's blue butterfly in our plots. For analyses of *Lupinus* responses to treatments, cover was determined by measuring the approximate rectangular area (length and width to the nearest cm) occupied by a clump. For analysis of community responses to treatments, cover of all other species was visually estimated in June 2009 to the nearest 1%; species with less than 1% cover were given a value of 0.1%. Total cover for a plot was at least 100%, and often exceeded that if many layers of vegetation were present. Species nomenclatures, provenance, and duration followed the USDA Plants database [31].

### Lupinus analyses

Due to the differences in past and current management at our sites, we tested for fertilizer treatment effects at each site separately. Phosphorus and M were used as fixed factors in all analyses; with two levels of each factor (present/not present). Cover of *Lupinus* was arcsin transformed and analyzed using a General Linear Model (SPSS 17.0, 2008) with cover in 2006 as a covariate. We also used a General Linear Model (SPSS 17.0, 2008) to analyze treatment effects on the number of inflorescences m^−2^ of *Lupinus*, with inflorescences m^−2^ in 2006 as a covariate (except at Baskett Butte, where no inflorescences were produced in 2006). Due to the non-normality of the data from Baskett Butte, at this site, we tested for differences in the rank abundances of the number of inflorescences m^−2^ using a General Linear Model (SPSS 17.0, 2008). We used a Generalized Linear Model (SPSS 17.0, 2008) with a negative binomial distribution to analyze treatment effects on the count of leaves, using the number of leaves in 2006 as a covariate; the response of leaves generally followed that of cover (see Tables S1, S2) and thus is not reported here.

### Community analyses

Although relatively minor components of the community initially, over time we observed increases in several non-native Fabaceae species, including *Vicia sativa* L., *V. hirsuta* (L.) Gray and *Trifolium dubium* Sibth. In our analyses, we considered all non-*Lupinus* legumes together, hereafter termed ‘other Fabaceae’. No other native Fabaceae species were present in the plots. We used a General Linear Model (SPSS 17.0, 2008) to test for a fertilizer effect on arcsin transformed cover of other Fabaceae.

We used multi-response permutation procedure (MRPP) to test for community responses to fertilizer treatments using PC-ORD 6 [32]. MRPP is a nonparametric permutation test that compares groups based on plot dissimilarity. The effect size, or *A-*statistic, reflects the degree to which groups differ and is independent of sample size [33]. We used Sørensen distances to calculate community dissimilarity. Analyses utilized 1000 Monte Carlo simulations in PC-ORD 6 [32]. Due to the potentially strong effect of the cover of *Lupinus* on these analyses (plots were selected to have relatively high cover of *Lupinus*), we conducted two analyses, first using the absolute cover of all species within the plots, and second using the relative cover of all species except *Lupinus* (thus removing the potential effect of *Lupinus*). When a significant treatment effect was found, we used an uncorrected multiple comparison test for differences between groups and indicator species analysis (utilizing 5,000 Monte Carlo simulations) to test association of species within treatment groups.

### Plant and soil nutrients

We determined plant tissue N concentrations and natural abundance δ^15^N stable isotope composition on four species common to all sites at the end of the 2008 growing season. In each plot, we collected entire aboveground tissues of 3–4 individuals each of *Fragaria virginiana*, *Plantago lanceolata* and *Vicia* spp, (*V. sativa* and *V. hirsuta* were sampled together as at the time of sampling, the species were indistinguishable) and leaves from 3–4 *Lupinus*, and composited samples by individual species. Samples were dried at 65°C for 48 hrs, ground to fine powder, and analyzed for%N and δ^15^N on a Thermo Electron Delta plus Advantage mass spectrometer with a Costech ECS-4010 elemental analyzer combustion at the Colorado Plateau Stable Isotope Laboratory. We used a fully-factorial ANOVA (SPSS 17.0; 2008) to evaluate effects of fertilization on plant tissue N and δ^15^N separately for each species. We also used least-squares linear regression (SPSS 17.0; 2008) of plot-level data to assess species-specific relationships between tissue N and δ^15^N.

We hoped to use δ^15^N data of putative N-fixers (*Lupinus* and *Vicia* spp) and the average δ^15^N of non-fixers (*Fragaria virginiana*, *Plantago lanceolata*) to calculate the percent of N derived from fixation (NDFA) by assuming that N-fixation occurred at the δ^15^N = 0‰ value of atmospheric N_2_ gas [34]. However, across all treatment and site combinations, our calculated NDFA for *Lupinus* violated method assumptions by significantly exceeding 100% in 1 of 12 possible cases, and the NDFA for *Vicia* spp. was significantly less than 0% in 1 out of 10 possible cases for which this species was present (one-sample t-tests). *Lupinus* spp. grown on N-free media in the greenhouse and forced to obtain N solely from fixation can display leaf and shoot δ^15^N ranging from −1.6‰ to + 4.2‰ depending on growth conditions, growth stage, degree of nodulation, and specific strain of rhizobial inoculum [35]–[38], and this range suggests that the assumption of fixed N = 0‰ could be violated. Likewise, assuming that non-fixing reference species adequately sample soil N available for putative N-fixers is always contentious, and our finding that *Vicia* spp. in one control plot were significantly more depleted than reference species suggestions this assumption may have been violated. We therefore rely on δ^15^N values alone to examine potential differences in N fixation among species.

We collected three mineral soil samples in each plot by coring (2 cm diameter, 0–10 cm depth) and created one composite soil sample per plot at the end of the 2008 growing season. Soils were sieved to 2 mm in the laboratory, and extracted within 24 hours of collection for inorganic N and P and within 48 hours for pH. Inorganic N was extracted by adding 35 mL of 2M KCl to 7 g field-moist soil, shaking for 1 h, then filtering by pouring through pre-rinsed Whatman 42 filter paper, followed by analysis for ammonium (NH_4_^+^) and nitrate (NO_3_^−^) on a Lachat QuikChem 8000 flow-injection autoanlyzer using the salicylate and cadmium reduction methods, respectively (QuikChem Methods 12-107-06-2-A and 12-107-04-1-F, Lachat Instruments, Milwaukee, WI, USA). Soil inorganic P was extracted by adding 25 mL of Bray-1 extracting solution (0.03 N NH_4_F-0.025 N HCl) to 5 g field-moist soil in 50 mL centrifuge tubes, shaking vigorously for 1 min, centrifuging at 3400 rpm for 5 min, filtering through Whatman 42 filter paper, followed by analysis using the molybdenum blue method (QuikChem Method 12-115-01-1-A, Lachat Instruments, Milwaukee, WI, USA). Soil solution pH was determined by mixing 20 mL deionized water with 10 g field-moist soil (2 water: 1 soil), allowing the mixture to equilibrate for 30 min, then measuring pH of the supernatant with an Accumet pH meter (Fisher Scientific, Hampton, NH, USA). We assayed for potential N mineralization in mineral soil by 28 day laboratory incubation of 10 g soil at 25 °C and 60% water holding capacity, followed by extraction and analysis for ammonium and nitrate as above, corrected for initial inorganic N. Gravimetric soil moisture content of these samples was determined by comparing the weight of a 10 g subsample before and after drying at 105°C for 48 hr. We also determined total C and N concentrations in control plots. 20 g of sieved soil were dried at 65°C for 48 hr, ground to a fine powder, then analyzed on a Costech ECS-4010 elemental combustion analyzer (Costech Analytical, Valencia, CA, USA). We used factorial ANOVA (SPSS 17.0; 2008) to evaluate effects of fertilization treatments on soil chemical variables.

## Results

### Response of *Lupinus*

**Figure 1:**
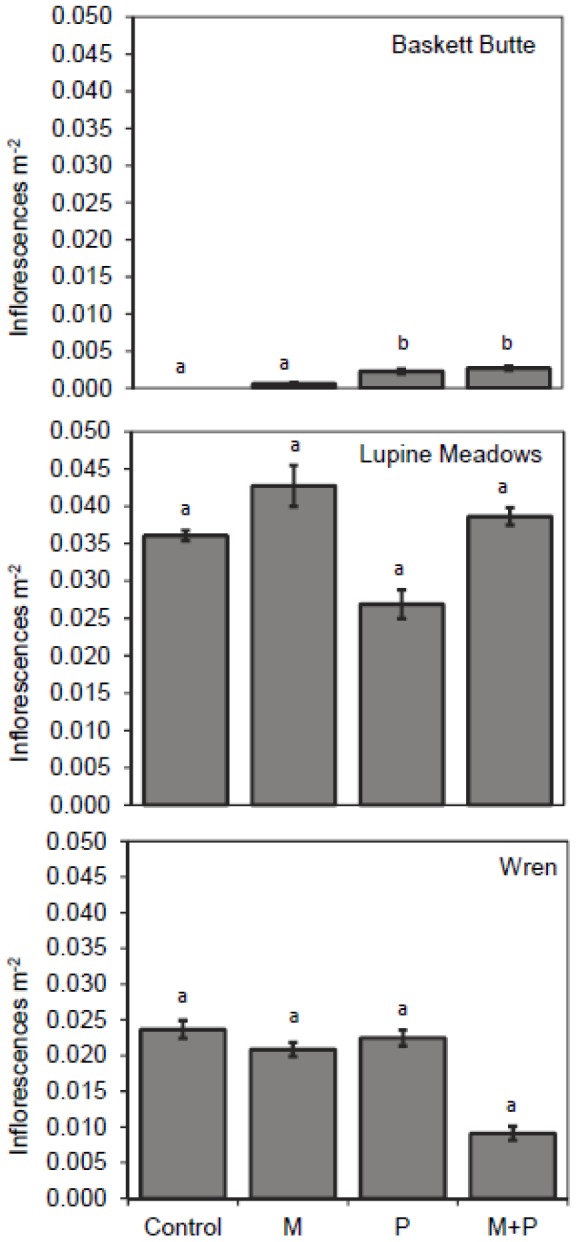
Mean number of *Lupinus* inflorescences m^−2^ lupine cover in plots treated with micronutrients and/or phosphorus. Treatments were applied at 3 sites in western Oregon. Bars are means±1 S.E.; n = 5 per treatment. There were effects of P at Baskett Butte (*P* = 0.063). Letters denote differences between treatments within species.

**Figure 2:**
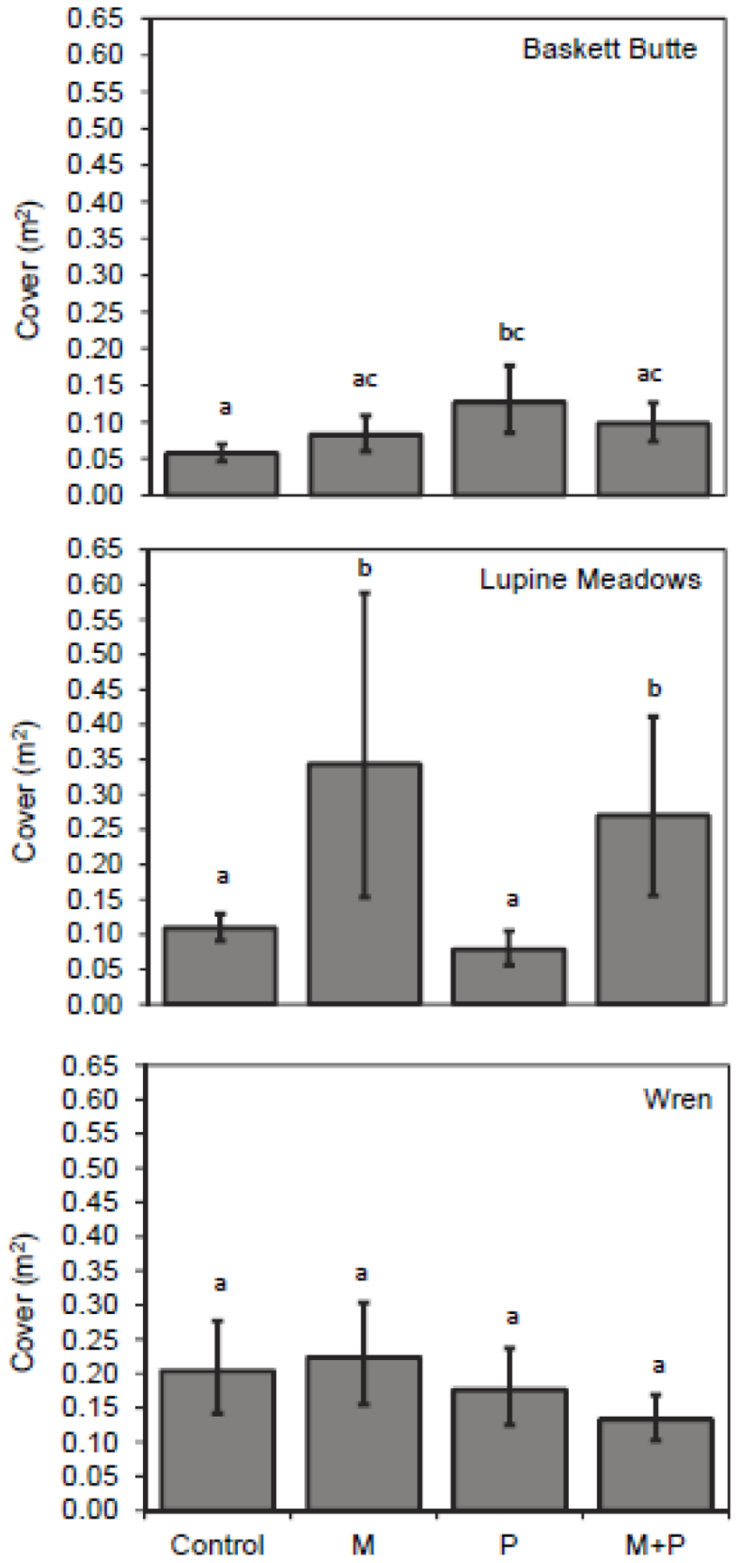
Cover of *Lupinus* in plots treated with micronutrients and/or phosphorus. Treatments were applied at 3 sites in western Oregon. Bars are means±1 S.E.; n = 5 per treatment. There were effects of P at Baskett Butte (*P* = 0.064) and M at Lupine Meadows (*P* = 0.057). Letters denote differences between treatments within species.

At Baskett Butte, P addition significantly increased *Lupinus* inflorescences (Figure 1; F_P_ = 3.846, df = 1,16, *P_P_* = 0.068; subscripts denote factors/levels); no inflorescences were produced in plots that received neither M or P. There was also an increase in cover (Figure 2; F_P_ =  3.989, df = 1,15, *P_P_* = 0.063) of *Lupinus* in plots treated with P. There were no effects of the M treatments, alone or in combination with P, on any of the *Lupinus* variables at this site (Figure 1, 2; see Tables S2, S3).

Treatment with M increased *Lupinus* cover (Figure 2; F_M_ =  0.318, df = 1,15, *P_M_* =  0.057) at Lupine Meadows. While there was a trend for increased inflorescence production in plots treated with M, this effect was not significant (Figure 1; F_M_ = 0.476, df 1,15, *P_M_* = 0.501). There were no effects on *Lupinus* of P addition and no significant interactions between P and M at this site (Figures 1, 2; see Tables S2, S3).

There were no treatment effects on cover or inflorescence production at Wren (Figures 1, 2, see Tables S2, S3).

### Community Response

**Figure 3:**
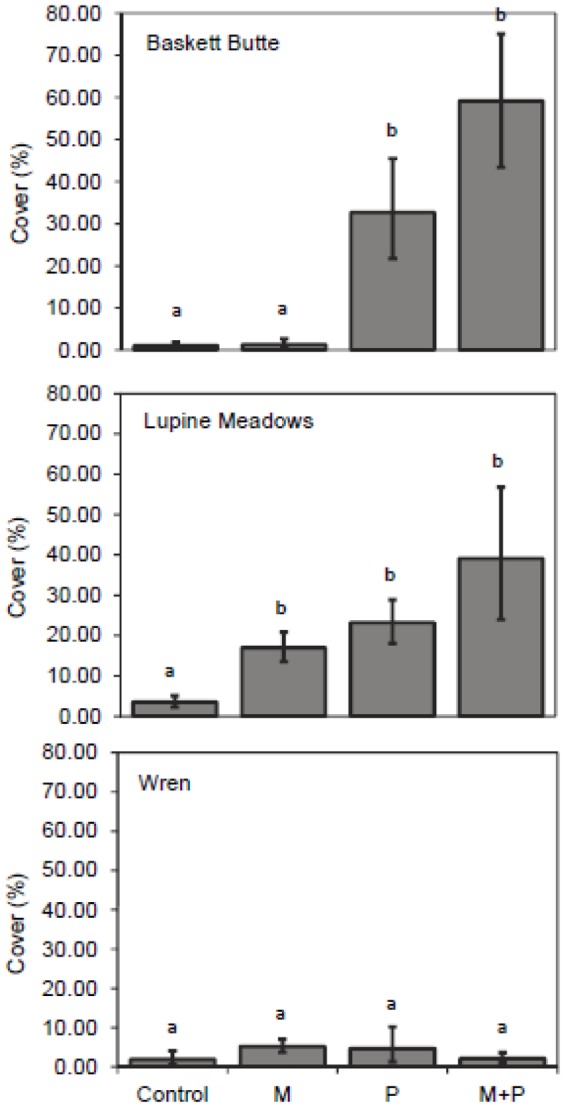
Cover of other Fabaceae in plots treated with micronutrients and/or phosphorus. Bars are means±1 S.E.; n = 5 per treatment. Treatment effects were significant for P at Baskett Butte (*P* <0.0005) and both M (*P* = 0.036) and P (*P* = 0.006) at Lupine Meadows. Letters denote differences between treatments within species.

The response of other Fabaceae to fertilizer treatments differed by site (Figure 3). Phosphorus alone increased the cover of other Fabaceae (F_P_ =  42.76, df = 1,16, *P_P_*<0.0005) at Baskett Butte. At Lupine Meadows, both M and P fertilizer treatments increased cover of other Fabaceae (F_M_ =  5.222, df = 1,16, *P_M_* =  0.036; F_P_ =  9.90, df  = 1,16, *P_P_* = 0.006). Finally, there was no effect of fertilizer treatments on other Fabaceae cover at Wren, and there were no treatment interactions on other Fabaceae at any of the sites (Table S2).

Using MRPP, we found no fertilization effects on community composition at our sites when all species were included in the analyses (Baskett Butte, A = −0.0555, *P* = 0.786; Lupine Meadows, A = −0.0064, *P*  = 0.901; Wren, A = 0.0262, *P* = 0.139). However, when we tested for treatment effects on the relative cover of all species except *Lupinus*, we did not find a treatment effect on community structure at Baskett Butte (A = 0.0382, *P* = 0.115) or Wren (A = −0.0020, *P* = 0.498), but there was a significant effect at Lupine Meadows (A = 0.0847, *P* = 0.004). Control plots differed from plots treated with M (A = 0.0983, *P* = 0.008), P (A = 0.1147, *P* = 0.007), and M+P (A = 0.1498, *P* = 0.004). *Leucanthemum vulgare* was associated with plots treated with P (IV = 36.2, *P* = 0.0116) and *Vicia hirsuta* was associated with plots treated with M+P (IV = 49.3, P = 0.029). There were no other significant indicator species.

### Plant and soil nutrients

In control plots, we found consistent differences among N-fixing and non-N-fixing species in tissue%N and δ^15^N at all sites (see Table S4). *Lupinus* had significantly higher tissue %N and more enriched δ^15^N than other species. *Vicia* spp. δ^15^N differed, but tissue %N did not, from non-N-fixing species at Wren and Lupine Meadows (*Vicia* spp. were not present in control plots at Baskett Butte thus precluding comparisons). Non-N-fixing *Plantago* and *Fragaria* did not differ significantly from one another in either tissue %N or δ^15^N at any site.

**Figure 4:**
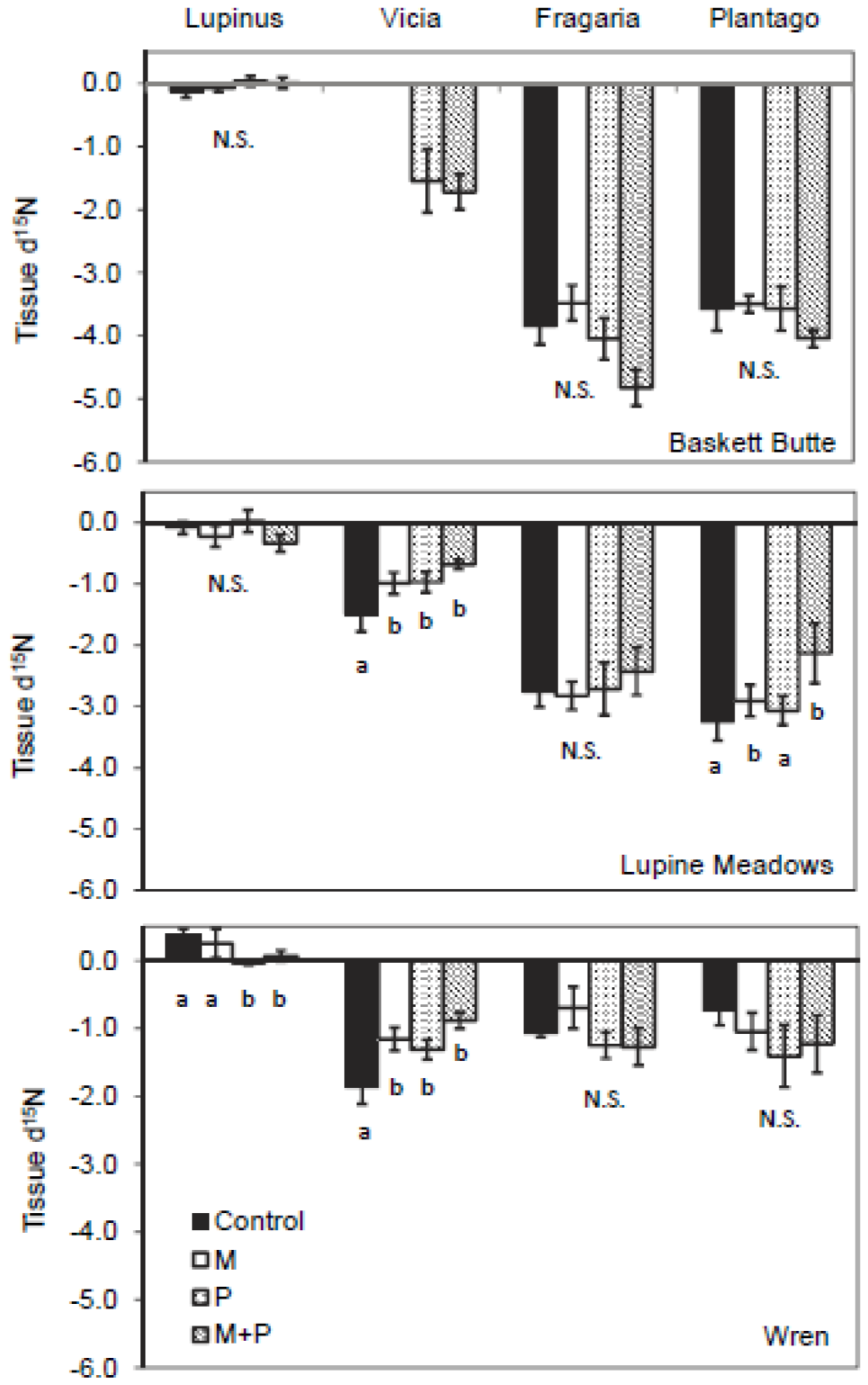
δ^15^N of *Lupinus, Vicia, Frgaria*, and *Plantago* in plots treated with micronutrients and/or phosphorus. Bars are means±1 S.E.; n = 5 per treatment (No *Vicia* were present in control and M plots at Baskett Butte). Letters denote differences between treatments within species. N.S. indicates where no differences were found. Treatment effects were not tested on *Vicia* at Baskett Butte as we were not able to obtain tissue samples within the control and M plots.

**Figure 5:**
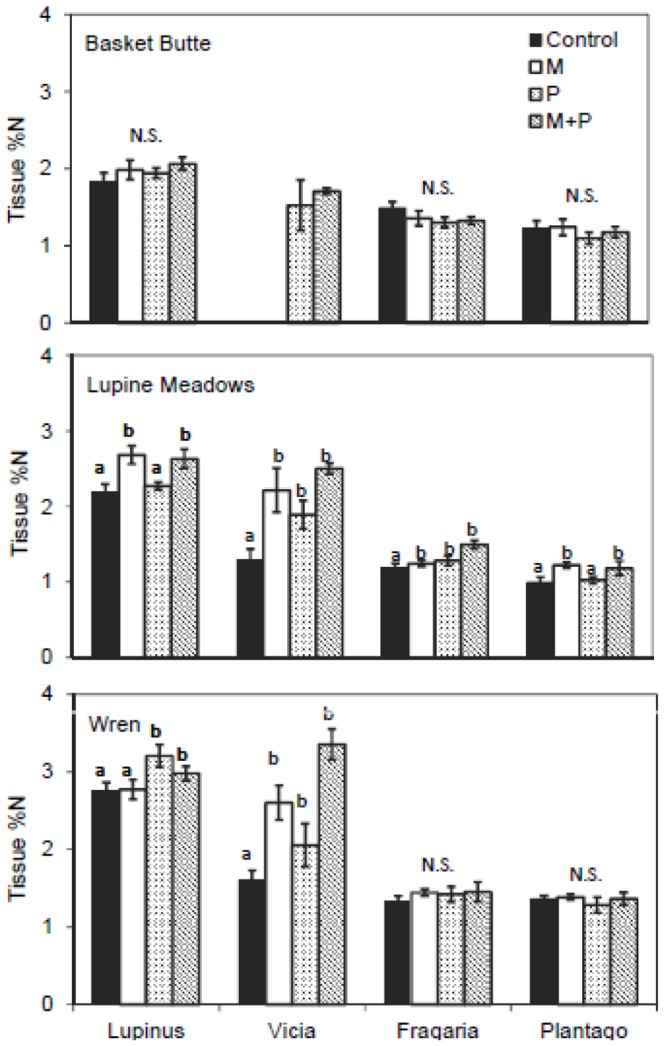
Tissue %N of *Lupinus, Vicia, Frgaria*, and *Plantago* in plots treated with micronutrients and/or phosphorus. Bars are means±s1 S.E.; n = 5 per treatment (No *Vicia* were present in control and M plots at Baskett Butte). Letters denote differences between treatments within species. N.S. indicates where no differences were found. Treatment effects were not tested on *Vicia* at Baskett Butte as we were not able to obtain tissue samples within the control and M plots.

Across all possible sites and treatments (n = 12), *Lupinus* δ^15^N did not differ significantly from 0‰ in 11 out of 12 cases, but control plots at Wren (0.3693‰) were significantly higher than 0‰ (p = 0.02, one-sample t-test). *Vicia* spp. were present in 10 of 12 possible site and treatment combinations, and displayed δ^15^N significantly less than 0‰ in 9 of 10 possible cases (except P treatment at Baskett Butte, *P* = 0.09). At Baskett Butte, we did not detect a significant effect of fertilization on δ^15^N or tissue %N in *Lupinus* (Table 2; Figures 4, 5). As *Vicia* spp. were present only in plots treated with P alone and in combination with Mat this site, this precluded an analysis of fertilization effects on tissue %N and δ^15^N.

**Table 2:** Tissue %N and δ^15^N of N_2_-fixing (*Lupinus* and *Vicia*) and non-N_2_-fixing (*Fragaria* and *Plantago*) species in control plots.

Site	Tissue %N				Tissue δ^15^N			
	*Lupinus*	*Vicia*	*Fragaria*	*Plantago*	*Lupinus*	*Vicia*	*Fragaria*	*Plantago*
Lupine Meadows	2.18^a^ (0.11)	1.29^b^ (0.14)	1.19^b^ (0.06)	0.99^b^ (0.07)	−0.08^a^ (0.098)	−1.50^b^ (0.287)	−2.76^c^ (0.24)	−3.25^c^ (0.30)
Baskett Butte	1.82^a^ (0.12)	–	1.49^b^ (0.08)	1.23^b^ (0.09)	−0.13^a^ (0.09)	–	−3.85^b^ (0.28)	−3.58^b^ (0.33)
Wren	2.75^a^ (0.12)	1.60^b^ (0.13)	1.33^b^ (0.07)	1.36^b^ (0.04)	0.37^a^ (0.10)	−1.87^b^ (0.25)	−1.08^c^ (0.05)	−0.74^c^ (0.22)

Notes: Data are averages with standard errors in parentheses. Dashes indicates that *Vicia* was absent from control plots at Baskett Butte. Significant differences among species within sites were analyzed using one-way ANOVA. Values in rows for tissue %N and δ^15^N that are followed by different letters are significantly different (p<0.10) by Tukey post-hoc comparison.

At Lupine Meadows, we detected a significant effect of M fertilization on *Lupinus* tissue %N (F_M_ =  16.54, df = 1,16, *P_M_*<0.001; Table 2), which increased from 2.18% N in control plots to 2.68% N in treated plots (Figure 5). We detected significant increases in *Vicia* spp. tissue %N in M and P fertilized plots (F_M_ =  16.16, df = 1,16, *P_M_*<0.001; F_P_ =  5.39, df = 1,16, *P_P_* =  0.034) that corresponded to shifts in *Vicia* spp. δ^15^N towards more atmospheric values (F_M_ =  4.27, df = 1,16, *P_M_* =  0.055; F_P_ =  4.67, df = 1,16, *P_P_* = 0.046). *Fragaria* tissue %N increased significantly in P and M treatments (F_M_ =  5.90, df = 1,16, *P_M_* =  0.027; F_P_ =  9.65 df = 1,16, *P_P_* =  0.007). *Plantago* responded slightly to M fertilization in both tissue%N (F_M_ =  3.75, df = 1,16, *P_M_* =  0.073) and δ^15^N (F_M_ =  3.95, df = 1,16, *P_M_* =  0.067), with values increasing in%N towards more atmospheric δ^15^N.

At Wren, significant shifts in tissue%N and δ^15^N due to fertilization occurred only in N-fixing species (Table 2; Figures 4, 5). For *Lupinus*, P fertilization significantly increased tissue%N (F_P_ =  7.75, df = 1,16, *P_P_* =  0.014) and slightly decreased δ^15^N towards more atmospheric values (F_P_ =  4.77, df = 1,16, *P_P_* =  0.045). In *Vicia* spp., both M and P significantly increased tissue%N (F_M_ =  29.04, df = 1,16, *P_M_*<0.001; F_P_ =  7.97, df = 1,16, *P_P_* =  0.012) and increased δ^15^N towards more atmospheric values (F_M_ =  10.31, df = 1,16, *P_M_* =  0.005; F_P_ =  5.44, df = 1,16, *P_P_* = 0.033).

**Figure 6:**
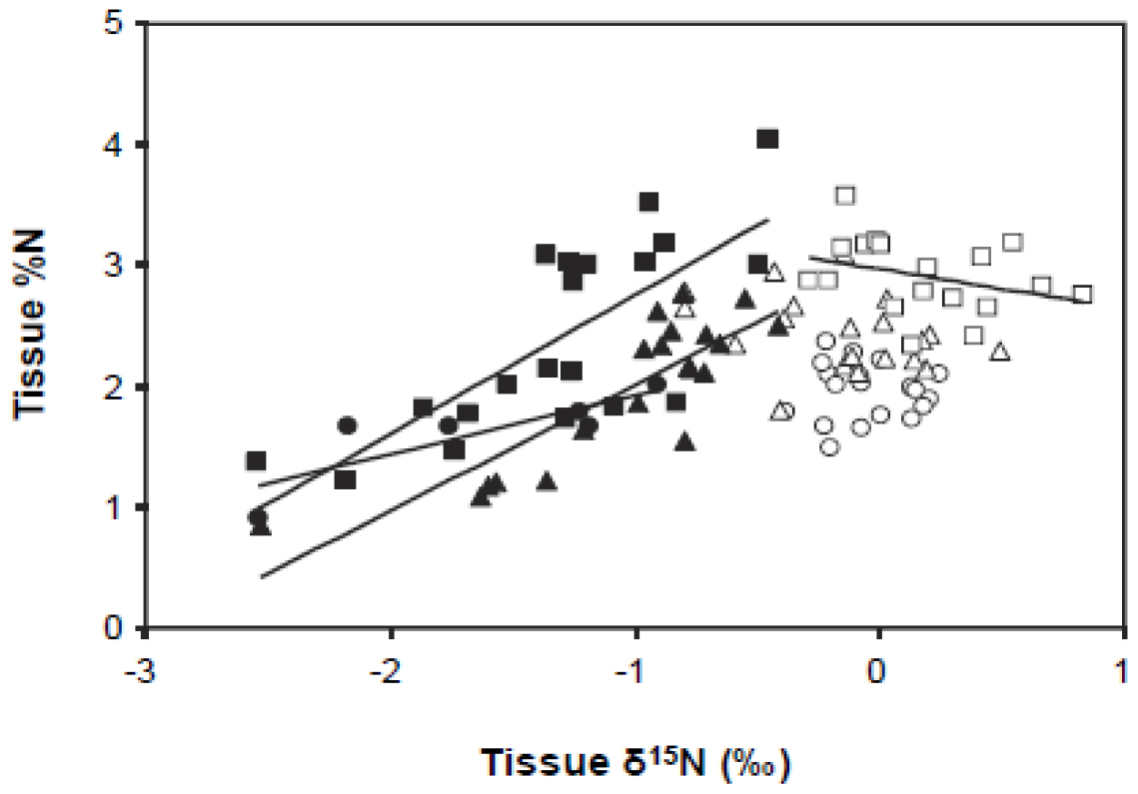
Relationship between tissue %N and δ^15^N for *Lupinus* and *Vicia* in all treatment plots. Filled symbols indicate *Vicia*, open symbols indicate *Lupinus*. Linear regressions are significant for *Vicia* at Baskett Butte (filled circles: R^2^ = 0.69, *P* = 0.04), Lupine Meadows (filled triangles, R^2^ = 0.73, *P*<0.001) and Wren (filled squares R^2^ = 0.56, *P*<0.001), and for *Lupinus* at Wren (open squaresR^2^ = 0.50, *P*<0.001).

When plot-level tissue data were evaluated by site, we found that *Vicia* spp. tissue%N consistently increased as δ^15^N shifted from depleted values towards 0‰ (Figure 6). For *Lupinus*, δ^15^N values overall were near 0‰, and there was only a weak linear relationship observed between tissue %N and δ^15^N at one site.

We observed very few fertilization effects on soil N and pH (data not shown). As expected, P fertilization increased soil extractable P at all sites, on average 10-fold over control and M-only plots. Soil NH_4_^+^ was marginally higher (F_P_ =  3.8, df = 1,16, *P*_P_ =  0.069) in P treated plots only at Lupine Meadows, and NO_3_^−^ did not differ among any treatments. Laboratory potential N mineralization was significantly lower (F_P_ =  12.1, df = 1,16, *P_P_* =  0.003) in P treated plots only at Baskett Butte. Soil pH was ∼0.2–0.6 units lower in M treated plots at all sites.

## Discussion

Our results demonstrate that non-N nutrients can limit N fixation and growth of threatened *Lupinus*. At sites where P or M promoted *Lupinus* reproduction or growth, the same nutrient generally increased *Lupinus* tissue N. *Lupinus* δ^15^N was consistently near the atmospheric value of 0‰ across all sites in both control and fertilized plots (0.03‰±0.14‰; mean±SE, n = 3 sites), whereas non-N-fixing reference species displayed depleted δ^15^N (*Fragaria*: –2.56‰±0.81; *Plantago*: –2.52‰±0.90), suggesting strong reliance of *Lupinus* on N-fixation as a source of N. Other *Lupinus* species growing on low-N soils can also show strong reliance on atmospheric N [39], although elevated N availability can shift *Lupinus* N acquisition towards soil N [40]. Due to the relative constant and small differences in δ^15^N values of *Lupinus*, these data can provide only suggestive evidence of N fixation patterns. Additional data on nodule biomass and nitrogenase enzyme activity would have been helpful, but unfortunately the federally threatened status of *Lupinus* at our field sites precluded more destructive soil sampling required for such measurements.

The apparent reliance of *Lupinus* on fixed N in our study plots likely reflects low ambient soil N availability, as suggested by low concentrations of total N, low potential net N mineralization, and low extractable NO_3_^−^ relative to NH_4_^+^ in surface mineral soils (Table 1). These low-N soils contrast sharply with the unusually N-rich soils of the coastal forest province bordering the Willamette Valley [41], and suggest that aboriginal burning of prairies [42], [43] likely maintained low-N soil conditions that favored N-fixers such as *Lupinus* in upland prairies. It is unclear whether recent increases in atmospheric N deposition in the Willamette Valley have altered N dynamics in vascular plant communities that include *Lupinus*, as has been observed for arboreal lichen communities in the area, which display increased thallus N, shifts in community composition towards nitrophilous species, and loss of N-fixing cyanolichens [44].

Non-native annual *Vicia* spp. also showed nutrient limitation of N fixation and growth, but in ways that differed from native, perennial *Lupinus*. At two of the sites (Baskett Butte and Lupine Meadows), *Vicia* spp. displayed δ^15^N values intermediate between atmospheric N_2_ and non-fixing species, and at the third site (Wren) δ^15^N overlapped with non-fixers. *Vicia* spp. also showed less site-specificity in response to fertilization treatments than *Lupinus*, responded to both M and P in the two sites where it was present in all plots, and was present only in P and M+P plots at the third site. In addition, the concomitant increase in tissue N with a shift towards atmospheric δ^15^N across all study plots (Figure 6) provides evidence for N limitation of *Vicia* spp., and suggests that constraints on N-fixation limit *Vicia* spp. tissue N to low values in these low-N sites. Thus, both N and non-N nutrients may be more important in shaping success of non-native *Vicia* spp. than native *Lupinus* in these sites. The relatively plastic reliance of *Vicia* spp. on soil versus atmospheric sources of N further suggests a potential to respond rapidly to variation in factors controlling N supply. This is consistent with evidence from agricultural settings showing that high N additions can suppress N-fixation in this genus [45]. Thus, non-N fertilization that increases N-fixation in invasive *Vicia* species is likely to lead to significant increases in growth and reproduction.

We found evidence that background levels of soil P, both alone and interactively with soil N, shaped patterns of nutrient limitation observed in *Lupinus*. At two sites with low soil P - Baskett Butte and Wren – we found significant effects of P fertilization on *Lupinus*. However, responses to P fertilization varied with background soil N availability. At Wren, where both soil P and soil N mineralization were low, P fertilization significantly increased *Lupinus* tissue N and changed tissue δ^15^N towards atmospheric values. At Baskett Butte, where soil P was low and soil N mineralization was high, P fertilization did not change tissue N or δ^15^N but did significantly increase *Lupinus* growth and reproduction. It is thought that P may limit either N-fixation or growth in symbiotic N fixing organisms [46]. Our results raise the possibility that the mode of P limitation may depend on N supply, whereby P primarily limits N fixation when soil N is scarce, but limits N-fixer growth when soil N is abundant.

Higher background soil P at Lupine Meadows appeared to alleviate P limitation, and only micronutrient fertilization increased *Lupinus* growth and tissue N. The higher levels of soil P and resulting micronutrient limitation of *Lupinus* at Lupine Meadows may reflect site management history; Lupine Meadows is the most recently grazed site, and grazing can preferentially increase the availability of P relative to other nutrients [47], [48]. Slightly lower soil pH at Lupine Meadows may have also decreased soil Mo availability [21], leading to stronger Mo limitation at this site, although future Mo-only fertilization is needed to conclusively identify which specific micronutrient stimulated *Lupinus* growth and tissue N at this relatively P-rich site. Indeed, species-specific access to and requirements for Mo, P and other non-N nutrients may contribute to the differential response of *Lupinus*, *Vicia* spp. and other Fabaceae to fertilization, and may constitute an important yet poorly understood control of symbiotic N_2_-fixation in many ecosystems worldwide [15], [49], [50].

The response of *Vicia* spp. to both M and P at our sites was surprising, as these elements are not substitutable in the biochemistry of N-fixation [51]. While this response may indicate co-limitation by these nutrients, we cannot exclude the possibility that the response of *Vicia* spp. to P fertilizer may also indicate a response to trace contamination of Mo in the P fertilizer. Analysis of our P fertilizer shows trace contamination with Mo of 9.2 mg Mo/kg (Julie Pett-Ridge, *personal communication*), within the range of typical triple-superphosphate fertilizers (2.4–18.5 mg Mo/kg) [52], resulting in low-levels of inadvertent Mo addition of 0.35 mg Mo m^−2^yr^−1^ in the P-only treatment. This represents only 1.7% of the Mo added deliberately in the M treatments and would be quite a sensitive response given that surface soil organic matter has high potential to immobilize Mo [53], whereas the symbiotic N_2_-fixing plants that we studied root and produce nodules in mineral soil. However, asymbiotic N fixation in surface organic soil can be stimulated by trace levels of Mo addition [10] and the soils that we studied are generally considered low in available Mo [22], so we cannot exclude it as a possibility.

The observed changes in the plant community suggest that increases in N fixation are primarily benefiting N-fixing species. At the site where fertilization significantly altered plant community composition (Lupine Meadows), the indicator species for this effect were the non-native non-N-fixing forb, *Leucanthemum vulgare*, and non-native N-fixer, *V. hirsuta*. The increase in *Leucanthemum* at Lupine Meadows was unexpected; future year's analyses should consider whether this species continues to respond to phosphorus fertilization. While we observed increased cover of non-native N-fixers in response to P fertilization at Baskett Butte, the lack of overall plant community response at this site is likely due to the relatively high variability in community composition between plots within the same treatment group (as indicated by an effect size close to 0; A = 0.0382). Greater shifts in plant community composition may not be apparent unless there is significant mortality of *Lupinus*, thus decreasing competition for light [54], [55]. While changes in plant species composition may take several years to occur, plant biomass may respond relatively quickly in response to fertilization [54]. Several treatment plots appeared visually to have higher biomass levels than control plots, and although we were not able to sample biomass due to the presence of the endangered Fender's blue butterfly, future measurements could include a surrogate for biomass, such as vegetation height.

The difference in the ability of the non-native *Vicia* spp. and native *Lupinus* to increase nitrogen fixation in response to nutrients may be related to differences in these species' life histories. *Lupinus* is a native, long-lived perennial forb that is generally found in relatively undisturbed remnant prairies. These prairies are expected to be relatively low in N, thus this species' long-term persistence in these communities likely depends on N-fixation. In contrast, the other Fabaceae species in our plots were exotic annual species. As these species are short-lived, they would benefit from being flexible and opportunistic in their strategy to obtain N. Our work adds to prior field studies that have demonstrated increased growth in leguminous species in natural ecosystems in response to P fertilization [17]–[20].

Five years of experimental fertilization with non-nitrogen fertilizers succeeded in increasing the growth and reproductive capacity of threatened *Lupinus* with few non-target plant-community effects. However, our results also suggest some caution in use of fertilization for recovery of rare N-fixing species, as we found both an apparently greater benefit to invasive nitrogen fixers to fertilization, as well as some site- and species-specificity of whether micronutrients or phosphorus fertilization were most effective. Such site specificity due to differences in management history and local abiotic conditions may contribute to the difficulties in identifying a generalized approach for successful restoration [56]. Background increases in atmospheric N deposition across the Pacific Northwest are poised to further increase the dominance of non-native grasses over native forbs, as has occurred in shrubland-grasslands of California [57]. It is unknown how such N inputs may interact with restorative non-N fertilization to shape community composition and other dynamics of prairie ecosystems. To the extent that changes in background N inputs, climate and other factors further threaten native plant communities in this region, it is possible that non-N fertilization may prove to be useful to maintain population levels of threatened legume species.

## Supporting Information

Figure S1 Locations of experimental sites
(PDF)

Table S1 Summary of tests of effects of micronutrient and phosphorus fertilizers on the number of *L. oreganus* leaves.
(PDF)

Table S2 Summary of two-way ANOVA of effects of micronutrient and phosphorus fertilizers on cover and inflorescences of *L. oreganus* and cover of other Fabaceae species.
(PDF)

Table S3 Summary of significant ANOVA tests of effects of micronutrient (M) and phosphorus (P) fertilizers on 2008 tissue %N and δ15N of *L. oreganus*, *Vicia* spp., *Fragaria*, and *Plantago*.
(PDF)
