# Supplementary material for: Nutrient Limitation of Native and Invasive N2-Fixing Plants in Northwest Prairies
Source: PLoS One. 2013 Dec 27;8(12):e84593. doi: 10.1371/journal.pone.0084593 (PMC3874015; doi:10.1371/journal.pone.0084593)
Supplement: Figure S1 — Locations of experimental sites (PDF) [file pone.0084593.s001.pdf]

Fertilizer experiment sites

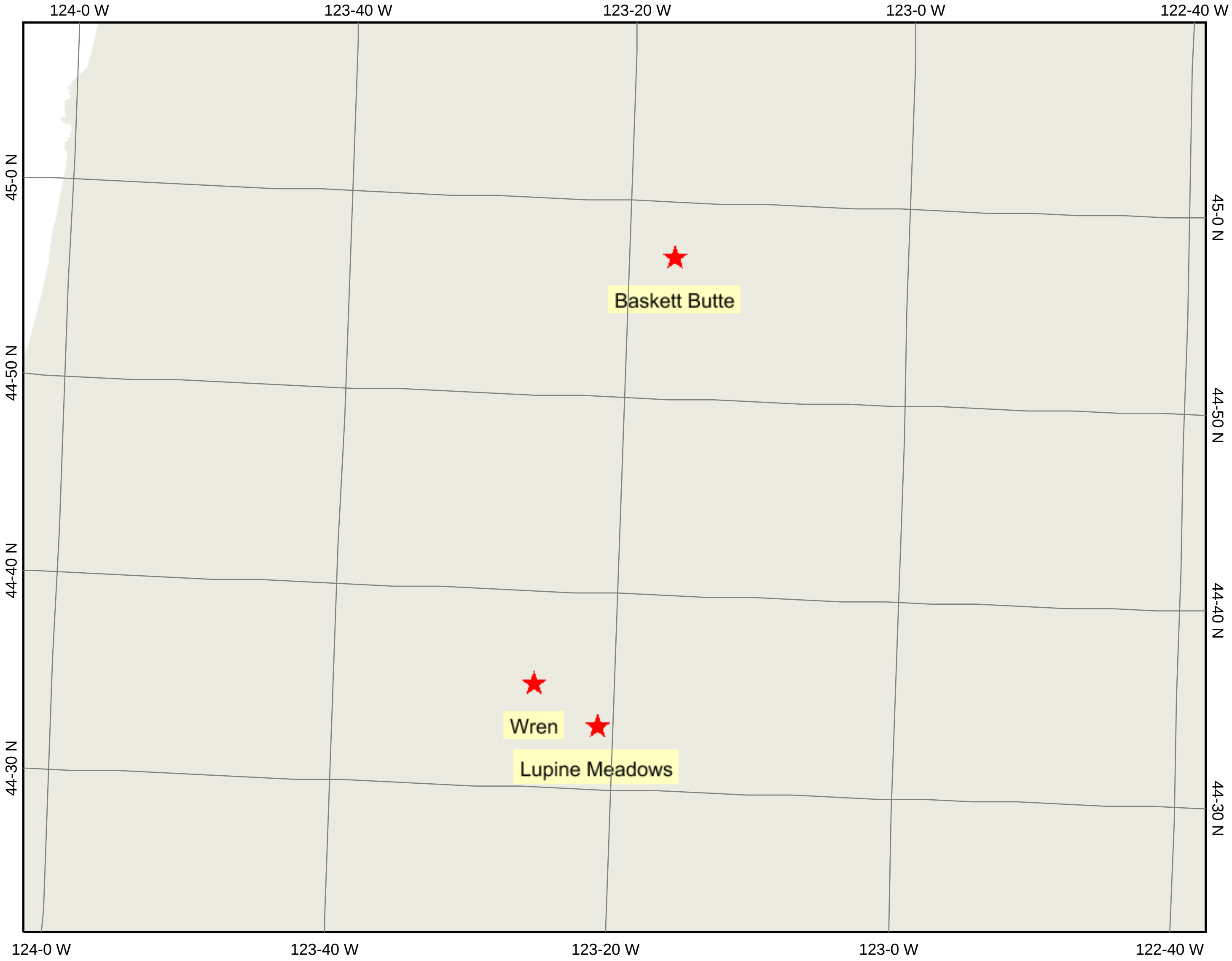

0 18 36 54 km.

Map center: 574430, 1111845

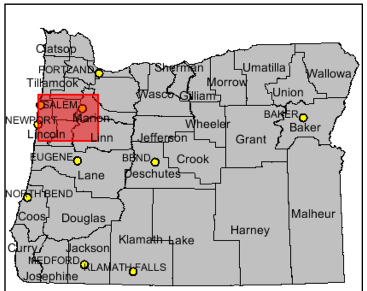

Legend

Scale: 1:600,000

This map is a user generated static output from Oregon Explorer (oregonexplorer.info) and is for general reference only. Data layers that appear on this map may or may not be accurate, current, or otherwise reliable. THIS MAP IS NOT TO BE USED FOR NAVIGATION.
