# Supplementary material for: Nutrient Limitation of Native and Invasive N2-Fixing Plants in Northwest Prairies
Source: PLoS One. 2013 Dec 27;8(12):e84593. doi: 10.1371/journal.pone.0084593 (PMC3874015; doi:10.1371/journal.pone.0084593)
Supplement: Table S1 — Summary of tests of effects of micronutrient and phosphorus fertilizers on the number of L. oreganus leaves. (PDF) [file pone.0084593.s002.pdf]

Table S1. Summary of tests of effects of micronutrient and phosphorus fertilizers on the number of *L. oreganus* leaves. P<0.10 are in bold.

Dependent variable	Factor	df	Wald Chi-Square	P
Lupine Meadows				
	Leaves 2006	1	0.231	0.631
	Phosphorus	1	0.310	0.577
	Micronutrients	1	8.432	**0.004**
	Phosphorus x Micronutrients	1	0.192	0.661
Wren				
	Leaves 2006	1	3.707	**0.054**
	Phosphorus	1	0.362	0.548
	Micronutrients	1	0.088	0.766
	Phosphorus x Micronutrients	1	0.091	0.763
Baskett Butte				
	Leaves 2006	1	3.811	**0.051**
	Phosphorus	1	0.384	0.535
	Micronutrients	1	0.665	0.415
	Phosphorus x Micronutrients	1	0.292	0.589

We used a Generalized Linear Model (SPSS 17.0, 2008) with a negative binomial distribution to analyze treatment effects on the count of leaves in 2009, using their respective counts in 2006 as covariates.
