# Supplementary material for: Nutrient Limitation of Native and Invasive N2-Fixing Plants in Northwest Prairies
Source: PLoS One. 2013 Dec 27;8(12):e84593. doi: 10.1371/journal.pone.0084593 (PMC3874015; doi:10.1371/journal.pone.0084593)
Supplement: Table S2 — Summary of two-way ANOVA of effects of micronutrient and phosphorus fertilizers on cover and inflorescences of L. oreganus and cover of other Fabaceae species. (PDF) [file pone.0084593.s003.pdf]

Table S2. Summary of two-way ANOVA of effects of micronutrient and phosphorus fertilizers on cover and inflorescences of *L. oreganus* and cover of other Fabaceae species. P<0.10 are in bold.

Dependent variable	Factor	Df	MS	F	P
Baskett Butte					
L. oreganus	Cover 2006	1	0.107	26.608	**<0.0005**
cover	Phosphorus	1	0.016	3.989	**0.064**
	Micronutrients	1	0.000	0.005	0.942
	Phosphorus x Micronutrients	1	0.010	2.514	0.134
	Error	15	0.004		
L. oreganus	Cover 2006				
inflorescences	Phosphorus	1	8.00×10^{-7}	4.000	**0.063**
	Micronutrients	1	2.00×10^{-7}	1.000	0.332
	Phosphorus x Micronutrients	1	0.000	0.000	1.000
	Error	16	2.00×10^{-7}		
other Fabaceae	Phosphorus	1	1.685	42.775	**<0.0005**
	Micronutrients	1	0.067	1.707	0.210
	Phosphorus x Micronutrients	1	0.052	1.333	0.265
	Error	16	0.039		
Lupine Meadows					
L. oreganus	Cover 2006	1	0.065	0.867	0.367
cover	Phosphorus	1	0.015	0.203	0.659
	Micronutrients	1	0.318	4.249	**0.057**
	Phosphorus x Micronutrients	1	0.002	0.020	0.889
	Error	15	0.075		
L. oreganus	Cover 2006	1	1.223×10^{-5}	0.744	0.402
inflorescences	Phosphorus	1	1.802×10^{-5}	1.096	0.312
	Micronutrients	1	7.820×10^{-6}	0.476	0.501
	Phosphorus x Micronutrients	1	4.456×10^{-6}	0.271	0.610
	Error	15	1.644×10^{-5}		
other Fabaceae	Phosphorus	1	0.335	9.900	**0.006**
	Micronutrients	1	0.176	5.222	**0.036**
	Phosphorus x Micronutrients	1	0.007	0.209	0.653
	Error	16	0.034		
Wren					
L. oreganus	Cover 2006	1	0.202	16.902	**0.001**
cover	Phosphorus	1	0.025	2.116	0.166
	Micronutrients	1	0.000	0.012	0.915
	Phosphorus x Micronutrients	1	0.003	0.224	0.643
	Error	15	0.012		
L. oreganus	Cover 2006	1	3.251×10^{-6}	0.565	0.464
inflorescences	Phosphorus	1	1.010×10^{-5}	1.755	0.205
	Micronutrients	1	1.255×10^{-5}	2.180	0.160
	Phosphorus x Micronutrients	1	3.122×10^{-6}	0.542	0.473
	Error	15	5.757×10^{-6}		

other Fabaceae	Phosphorus	1	$1.5*10^{-5}$	0.001	0.980
	Micronutrients	1	0.001	0.025	0.876
	Phosphorus x Micronutrients	1	0.032	01.436	0.248
	Error	16	0.023		

Tests were performed using using a General Linear Model (SPSS 17.0, 2008). For *L. oreganus* analyses, cover and inflorescences in 2006 were used as a covariates in their respective analyses, except for inflorescences at Baskett Butte. No inflorescences were produced at Baskett Butte in 2006, thus this was not included as a covariate in the analysis.
