# Supplementary material for: Nutrient Limitation of Native and Invasive N2-Fixing Plants in Northwest Prairies
Source: PLoS One. 2013 Dec 27;8(12):e84593. doi: 10.1371/journal.pone.0084593 (PMC3874015; doi:10.1371/journal.pone.0084593)
Supplement: Table S3 — Summary of significant ANOVA tests of effects of micronutrient (M) and phosphorus (P) fertilizers on 2008 tissue %N and δ15N of L. oreganus, Vicia spp., Fragaria, and Plantago. (PDF) [file pone.0084593.s004.pdf]

Table S3. Summary of significant ANOVA tests of effects of micronutrient (M) and phosphorus (P) fertilizers on 2008 tissue %N and δ^{15}N of *Lupinus*, *Vicia* spp., *Fragaria*, and *Plantago*. Bold text indicates P < 0.05, plain text indicates P < 0.10, n.s. indicates not significant, and n.d. indicates no data.

**Site and Species**	**Significant Treatments for Tissue %N**	**Significant Treatments for Tissue δ^{15}N**
Lupine Meadows		
Lupinus	**M**	n.s.
Vicia spp.	**P, M**	**P**, M
Fragaria	**P, M**	n.s.
Plantago	M	M
Wren		
Lupinus	**P**	**P**
Vicia spp.	**P, M**	**P, M**
Fragaria	n.s.	n.s.
Plantago	n.s.	n.s.
Basket Butte		
Lupinus	n.s.	n.s.
Vicia spp.	n.s.	n.s.
Fragaria	n.s.	**P**, PxM
Plantago	n.s.	n.s.
